# Hepatocyte-derived exosomal MiR-194 activates PMVECs and promotes angiogenesis in hepatopulmonary syndrome

**DOI:** 10.1038/s41419-019-2087-y

**Published:** 2019-11-07

**Authors:** Lin Chen, Yi Han, Yujie Li, Bing Chen, Xuehong Bai, Karine Belguise, Xiaobo Wang, Yang Chen, Bin Yi, Kaizhi Lu

**Affiliations:** 1 Department of Anaesthesia, Southwest Hospital, The Third Military Medical University, Chongqing, China; 2 LBCMCP, ×tégrative (CBI), Université de Toulouse, CNRS, UPS, Toulouse, France

**Keywords:** Liver diseases, Respiratory tract diseases

## Abstract

Hepatopulmonary syndrome (HPS) is a serious vascular complication in the setting of liver disease. Factors produced by the liver are essential to regulate pulmonary angiogenesis in the pathogenesis of HPS; however, the pathogenic mechanisms of pulmonary angiogenesis are not fully understood. We investigated the role of HPS rat serum exosomes (HEs) and sham-operated rat serum exosomes (SEs) in the regulation of angiogenesis. We found that HEs significantly enhance PMVEC proliferation, migration, and tube formation. We further identified miR-194 was the most notably increased miRNA in HEs compared to SEs. Once released, hepatocyte-derived exosomal miR-194 was internalized by PMVECs, leading to the promotion of PMVEC proliferation, migration, and tube formation through direct targeting of THBS1, STAT1, and LIF. Importantly, the pathogenic role of exosomal miR-194 in initiating angiogenesis was reversed by P53 inhibition, exosome secretion inhibition or miR-194 inhibition. Additionally, high levels of miR-194 were found in serum exosomes and were positively correlated with P(A-a)O_2_ in HPS patients and rats. Thus, our results highlight that the exosome/miR-194 axis plays a critical pathologic role in pulmonary angiogenesis, representing a new therapeutic target for HPS.

## Introduction

Hepatopulmonary syndrome (HPS) is a progressive disease that is characterized by worsening hypoxemia due to intrapulmonary vascular dilatation (IPVD), arteriovenous malformations and increased angiogenesis in the setting of chronic liver disease^1–3^. The prevalence of HPS varies from 4% to 47% due to different cut-offs in defining arterial hypoxemia, and the mortality rate of HPS is ~41%^4–6^. Over the past two decades, the pathogenesis and precise mechanisms of HPS have been under active investigation. Based on experimental and clinical research, the mechanisms of HPS continue to be uncovered, which provides the ability to clearly understand HPS pathogenesis and identify potential therapeutic targets. Although progress has been made in delineating the mechanisms underlying the imbalance of vasoactive substances, pulmonary vascular alterations, and angiogenesis in HPS, to date, there is still a lack of related practical therapeutic approaches apart from liver transplantation^7,8^. Recently, several lines of evidence have suggested that the pathophysiology of HPS may involve other factors in addition to IPVD. Early studies found an increased pulmonary capillary density in the microvasculature during cirrhosis, and increasing numbers of recent studies suggest that angiogenesis plays an essential role in the pathophysiology of human and experimental HPS^9–11^.

Exosomes are nanometer-sized vesicles that range in size between 30 and 150 nm and are released by cells upon fusion of multivesicular bodies with the plasma membrane^12^. Exosomes can shuttle bioactive molecules, including proteins and nucleic acids, such as microRNAs, from one cell to another, resulting in the exchange of genetic information and reprogramming of recipient cells^13,14^. As agents of cell-to-cell communication, exosomes play important roles in many diseases, such as cancer, cardiovascular diseases and acute lung injury^15–21^. Recently, the recognition understanding that exosomes modulate the angiogenic process of endothelial cells has been expanding^22–24^. Thus, it is interesting and important to explore whether exosomes are involved in and critical to HPS. However, it is still unclear how exosomes are altered in the pathogenesis of HPS and whether this alteration is detrimental or beneficial to HPS.

Our previous research demonstrated that common bile duct ligation (CBDL) rat serum induces excessive proliferation, migration, and tube formation of PMVECs in vitro, which could contribute to HPS-associated angiogenesis^25–27^. However, which ingredients in serum are involved in angiogenesis should be further clarified. In the present study, we hypothesized that serum exosomes might play a pivotal role in the pathological alterations of HPS. To evaluate this hypothesis, we assessed whether exosome levels or exosome contents are changed in CBDL rat serum, and whether exosomes could affect the pathological status of HPS in in vivo and in vitro models.

## Method

### Animal model

Male Sprague-Dawley rats were obtained from the Animal Center of the Third Military Medical University (Chongqing, China). An experimental HPS rat model was successfully established by CBDL as previously described^28,29^. Rats were randomly divided into different groups (no blinding was done). Under isoflurane inhalation anesthesia, the HPS group underwent CBDL, while the control group underwent common bile duct exposure but no ligation. The SMase inhibitor GW4869 (15 mg/kg) (Sigma, USA), p53 inhibitor pifithrin-μ (15 mg/kg) (Sigma, USA), or miR-194 inhibitor anta-miR-194 (2 mg/kg) (GeneChem, China) was administered every 5 days for 5 weeks by intravenous injection following CBDL. Control group rats were injected with saline (0.9% NaCl) containing no drug. Four rats were excluded owing to complications during surgery. Specimens were collected at 5 weeks postoperatively. All rats were housed under standard laboratory living conditions (22–24 °C, 12 h light/12 h dark cycle) and were fed a standard laboratory diet (Altromin, Germany). All procedures performed on the animals were conducted according to the guidelines from the National Institutes of Health. In addition, all experimental protocols were approved by the ethical committee of Third Military Medical University.

### Cell culture

Primary hepatocytes were isolated and cultured from Sprague-Dawley rats (male, 4 weeks) according to a published protocol^30^. Kupffer cells were isolated with OptiPrep Density Gradient Medium (Sigma, USA) as previously described^31^. Stellate cells were isolated and purified by collagenase and pronase^32^. The purity of stellate cells was >90%, as determined by intrinsic vitamin A autofluorescence. Cultured rat PMVECs were isolated, purified, and cultured from healthy Sprague-Dawley rats as previously described^33^.

### Exosome isolation, characterization, and treatment

Exosomes were purified from hepatocytes or PMVEC-derived conditioned media or serum with or without HPS by ultracentrifugation. Hepatocytes and PMVECs were cultured in DMEM supplemented with 10% fetal bovine serum and 1% penicillin–streptomycin (Invitrogen). Exosomes in bovine serum were depleted by ultracentrifugation at 175,000 × *g* at 4 °C for 16 h prior to use. After the designated amount of time, conditioned media were collected and centrifuged at 500 × *g* for 10 min at 4 °C, followed by 16,800 × *g* for 30 min at 4 °C. The supernatant was filtered through a 0.22 μm filter (Millipore), followed by ultracentrifugation at 110,000 × *g* for 70 min at 4 °C. The exosome pellet was washed with calcium and magnesium-free phosphate-buffered saline (PBS), followed by a second ultracentrifugation at 110,000 × *g* for 70 min at 4 °C and then resuspended in PBS. The amount of exosomes was measured by the Bradford assay (Bio-Rad, USA).

### Serum collection from patients

HPS patient screening was performed from October 2017 and January 2018 at the Southwest Hospital of the Third Military Medical University in Chongqing, China. Serum was separated from the blood samples of patients newly diagnosed with HPS. The study was approved by the ethics committee of the Third Military Medical University and written informed consent was obtained from all subjects (ethics number: NCT03435406).

### Transmission electron microscopy

After washing in PBS, the exosomes were fixed in 1.5 M sodium cacodylate buffer (pH 7.4) and were absorbed onto Formvar/carbon support film copper-mesh grids and negatively stained with 2% (wt/vol) uranyl acetate. Samples were observed using a transmission electron microscope (TEM). Digital images were acquired with an AMT digital camera system.

### Exosome–PMVEC fusion

Exosomes were labeled with PKH67 (Sigma, USA) ex vivo for 5 min, washed and added to the culture of PMVECs for 24 h. The samples were then stained with DAPI and analyzed by fluorescence microscopy.

### miRNA expression profiling

The serum exosomes isolated from the control and HPS rats were lysed, and total RNA was extracted using a miRNeasy Micro Kit (QIAGEN, Germany) according to the manufacturer’s protocol. miRNA expression profiling was performed using the miRCURY LNA Array (Exiqon, Denmark) system. The miRNAs that were significantly different between the two groups were identified according to the *P*-value and fold change. Hierarchical clustering was performed to show the differential miRNA expression profiles among samples.

### Quantitative real-time PCR (qRT-PCR)

The microarray results were validated by qRT-PCR. qRT-PCR was also performed to detect mRNA and exosomal miRNA. The candidate miRNAs included aberrantly upregulated miRNAs from the microarray, which were detected to observe their levels in the different groups. The miScript RT II Kit (QIAGEN, Germany), miScript Primer Assay (QIAGEN, Germany), and miScript SYBR Green PCR Kit (QIAGEN, Germany) were used to perform qRT-PCR according to the manufacturer’s instructions. The Ct value of the PCR results was calculated.

### Luciferase reporter assay

HEK293T cells plated in a 48-well plate were cotransfected with 2 ng of pRL-TK (Promega, USA), 20 ng of a firefly luciferase reporter that included a wild-type or mutant 3′-UTR of a target gene, and 10 nM of miR-194 or NC mimics. At 48 h after transfection, the cell lysates were used in a luciferase assay according to the manufacturer’s instructions.

### Cell cycle analysis

Cell cycle analysis was undertaken using a Cell Cycle Detection Kit (Beckman Coulter, USA) following the manufacturer’s instructions as previously described^34^.

### Wound healing assay

PMVECs at 90–95% confluence were serum starved in six-well plates for 24 h and then carefully scratched using sterilized pipette tips. PMVEC movements were recorded using a DM IRE2 microscope (Leica, Germany) every 10 min for up to 12 h. Thirty cells were recorded in each field of view. Both the migration path and direction of PMVECs were imaged by time-lapse video microscopy and analyzed using Matlab software.

### Transwell migration assay

Transwell migration assays were performed using 24-well Transwell chamber plates (Corning, USA). PMVECs (1 × 10^5^ cells/200 μl per well) were seeded in the upper compartment of the transwell chamber. The lower compartment was filled with 0.5 ml of the cell culture medium. After 12 h of incubation, the PMVECs that migrated through the polycarbonate membrane were fixed, stained, and counted under a microscope equipped with a digital imaging system (Nikon, Japan).

### Endothelial tube formation assay

Matrigel (Corning, USA) was thawed at 4 °C overnight and then coated on the bottom of a 96-well plate (50 µl per well) at 37 °C for 1 h. PMVECs (1 × 10^4^ cells/200 μl per well) suspended in starvation medium were added to the matrigel and cultured at 37 °C and 5% CO_2_ for 12 h. Vessel tube-like structures were observed and photographed under a microscope with a digital imaging system (Nikon, Japan). Then, the data were analyzed using ImagePro Plus software.

### Western blotting

Samples in different groups were lysed by the RIPA buffer containing 1% protease inhibitor PMSF. The lysates were centrifuged and then supernatants were collected. Equal proteins were added on an 8–12% Bis–Tris gel and then transferred to a polyvinylidene difluoride membrane. After transfer, membrane was treated with blocking solution for 2 h and probed with primary antibody against P53 (Abcam, USA), CD9 (Abcam, USA), CD63 (Abcam, USA), CD81 (Abcam, USA), HSP70 (Abcam, USA), Ang-2 (CST, USA), VEGF (Novus, USA), THBS1 (Santa Cruz, USA), STAT1 (Novus, USA), and LIF (Abcam, USA) overnight at 4 °C, followed by horseradish peroxidase-conjugated secondary antibody for 1 h. The loading control was the constitutively expressed protein β-actin (Sigma, USA) or lamin B (Abcam, USA). The blots were visualized with enhanced chemiluminescence system and quantitated using ImageJ.

### Immunofluorescence

Five-micron sections from 10% formalin paraffin-fixed lung tissues were blocked and incubated with von willebrand factor antibody (Abcam, USA) followed by Alexa Fluor 555-labeled secondary antibody (Beyotime Inc., China). PMVECs were fixed with 10% formalin for 30 min, permeability with 0.3% Triton X-100 for 10 min and blocked with 10% goat serum for 1 h at room temperature. Cells were then incubated with phalloidin-rhodamine for 20 min for staining off-actin. DAPI was used for nuclear staining (Beyotime Inc., China). Micrographs were obtained with a fluorescent microscope (Olympus BX51, Japan).

### Cell transfections

miR-194 mimics, miR-194 inhibitor (Anti-miR-194), and their negative control (NC and Anti-NC, respectively) were purchased from Genechem (China). 10 nM of miR-194 mimics, miR-194 inhibitors, NC, and Anti-NC were transfected using Lipofectamine RNAiMAX (Invitrogen, USA) in serum-free medium following manufacturer’s instructions. For exosomes transfection, miR-194 inhibitors were loaded in exosomes using Exo-Fect Exosome Transfection Kit (System Biosciences, USA).

### Histological analysis

Lung and liver tissues were collected and fixed for histological analysis as previously described^35^. Briefly, after lung and liver tissues were fixed in 10% formalin for 24 h, dehydrated in alcohol, embedded in paraffin, cut into 5-μm thickness sections and stained with hematoxylin and eosin (H&E). The microphotographs of the specimens were obtained with a light microscope (Olympus, Japan).

### Enzyme-linked immunosorbent assay

The levels of Ang-2, VEGF in medium or lung tissue and ALB, HB in serum exosomes were measured by ELISA assay kits following the manufacturer’s instructions (R&D Systems, USA). After different treatments, the sample was collected after centrifugation at 1000 × *g* for 20 min and 100 μl of supernatant was used for detection. The absorbance was measured using a spectrophotometer at 450 nm. The concentrations were calculated from the standard curve and presented as pg/ml.

### Statistics

All data are expressed as the mean ± standard deviation of the mean (SD). Unless otherwise indicated, for all in vitro experiments, data from at least five independent experiments were analyzed. A two-tailed unpaired Student’s *t*-test was used for comparison between two groups, and one-way ANOVA were performed for comparisons of data with more than two groups followed by Bornferroni correction for multiple comparisons. Overall survival of the experimental group was analyzed by the Kaplan–Meier survival curve. Differences were considered statistically significant at *p* < 0.05. Sample size was chosen according to previous observations, which perform similar experiments to see significant results, or the results from our preliminary experiments. Variance was similar between the groups that are being statistically compared. All analyses were performed with SPSS version 19.0.

## Results

### Quantification of serum exosomes in controls and HPS model

**Fig. 1:**
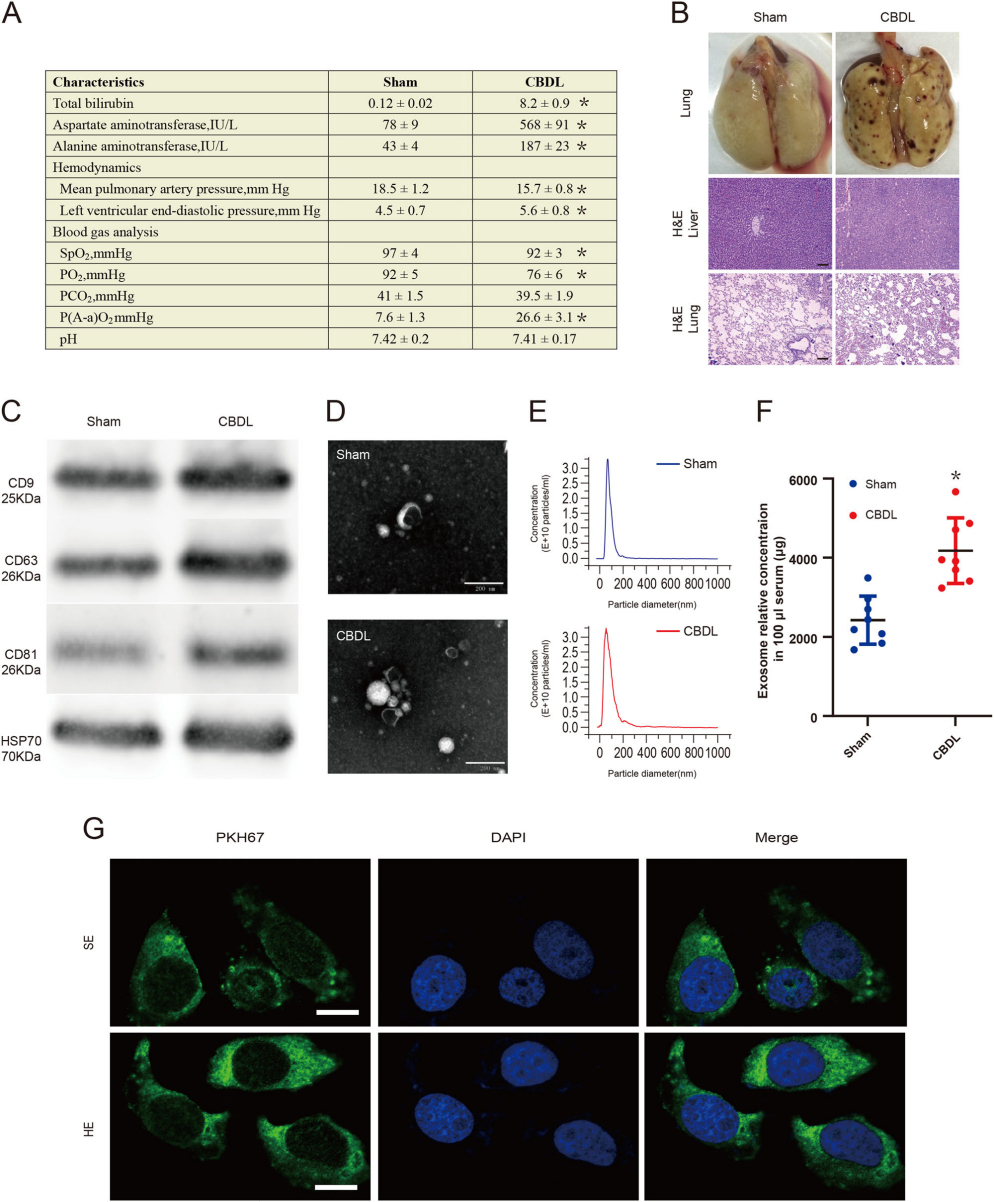
Characterization of exosomes derived from rat peripheral blood serum in the sham-operated group and CBDL group. **a** Comparison of liver function test results, hemodynamics and lung function test results in the sham-operated group and CBDL group (*n* = 10). **b** Comparison of histological features of injury in study groups (*n* = 10). Scale bar, 100 μm. **c** CD9, CD63, CD81, and HSP70 immunoblots of exosomes derived from rat peripheral blood serum in the sham-operated group and CBDL group (*n* = 5). **d** Transmission electron micrographs of exosomes derived from rat peripheral blood serum in the sham-operated group and CBDL group (*n* = 10). Scale bar, 200 nm. **e** The concentration and size of SEs and HEs were compared using NTA (*n* = 6). Data are represented as the means ± SD. *p < 0.05, compared with the sham-operated group. **f** The enriched exosomal protein population was quantified in rat blood serum from the sham-operated group or CBDL group using the BCA assay (*n* = 8). **g** The detection of exosome uptake by PMVECs in vitro. Exosomes are shown (PKH67 in green, DAPI in blue). Scale bar, 25 μm

To explore the changes of serum exosomes in the HPS model, HPS was induced by CBDL as previously reported^28^. As shown in Fig. 1a, the levels of total bilirubin, blood aspartate aminotransferase, alanine aminotransferase, and P(A-a)O_2_ were extremely higher but oxygen saturation and PaO_2_ were predominantly lower in the CBDL-treated rats when compared with the sham-operated rats (*p* < 0.05). Besides, it was revealed in Fig. 1b that the development of biliary cirrhosis and lung architectural changes could be confirmed by the histological analysis. The above results validated the availability of CBDL for establishing experimental HPS model.

After isolating from serums of CBDL or sham-operated rats by ultracentrifugation, exosomes were characterized by western blotting assay, TEM, and NTA. In Fig. 1c, western blot results indicated that the exosomal characteristic markers (CD9, CD63, CD81, and HSP70) were detected in these exosomes. Meanwhile, exosomes, with the diameter about 100 nm, exhibited typical sphere-shaped bilayer membrane structure observed by TEM as represented in Fig. 1d. The concentrations and sizes of SEs and HEs of different groups were compared based on NTA. Figure 1e showed that the peaks of particle sizes were ~100 nm. Additionally, the concentration of HEs was markedly higher than that of SEs (*p* < 0.05), which was displayed in Fig. 1f.

To examine whether serum exosomes could be transferred to PMVECs, PMVECs were incubated with PKH67-labeled exosomes, and the uptake of exosomes was visualized by confocal microscopy. As displayed in Fig. 1g, when incubated with PMVECs, serum exosomes labeled with fluorescent PKH67 were internalized by unstained PMVECs over time. Interestingly, under our experimental conditions we were able to detect exosomes transfer into PMVECs in vivo (Supplementary Fig. S1).

### Exosomes promote PMVECs proliferation, migration, and tube formation

**Fig. 2:**
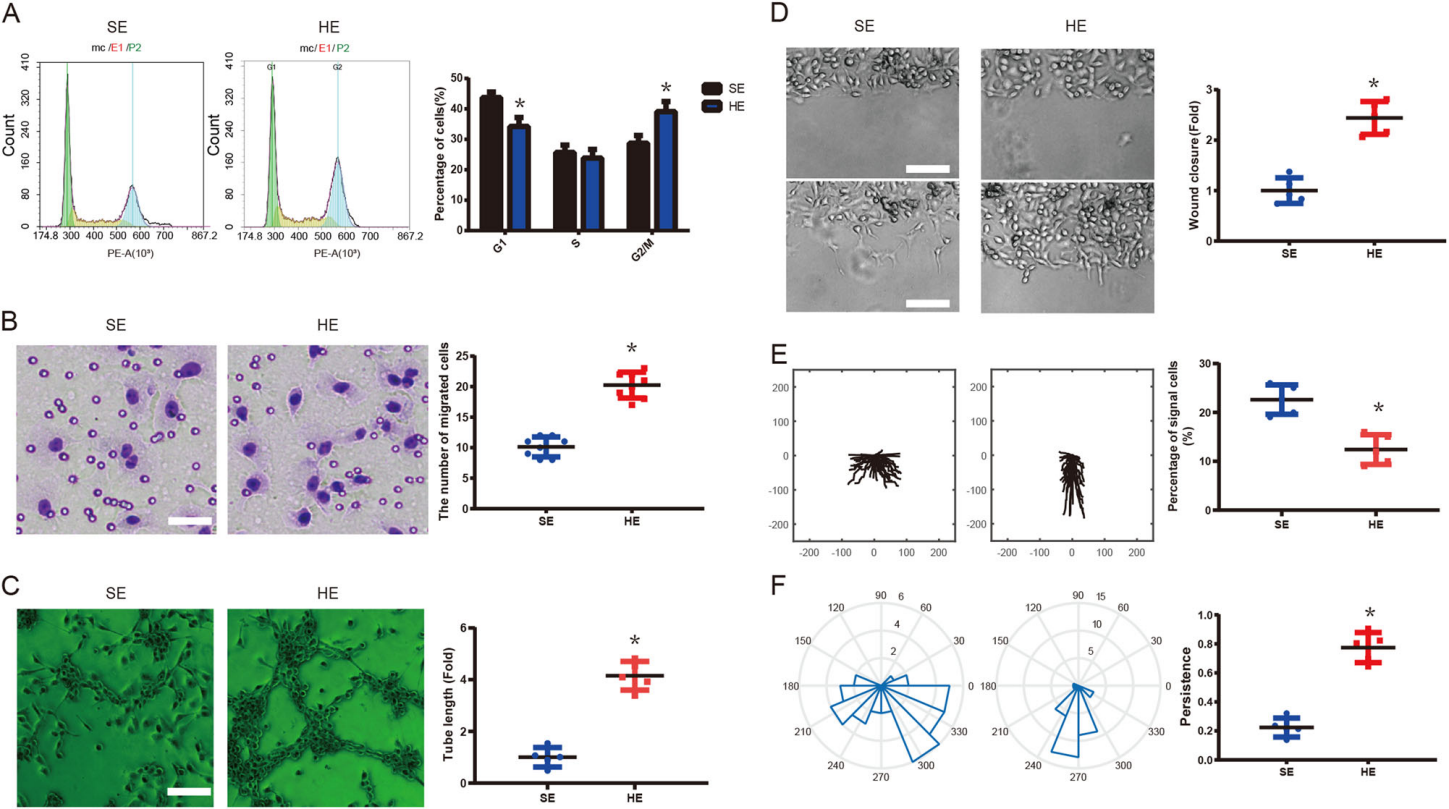
Effects of serum exosomes on PMVEC proliferation, migration, and tube formation. **a** Flow cytometric analysis was performed to detect cell cycle progression after incubation with SEs or HEs for 24 h (*n* = 6). **b** Transwell migration assay of PMVECs after incubation with SEs or HEs for 24 h (*n* = 8). Scale bar = 50 μm. **c** Tube formation assay of PMVECs after incubation with SEs or HEs (*n* = 5). Scale bar = 200 μm. **d** Wound healing assay of PMVECs after incubation with SEs or HEs for 24 h (*n* = 5). Scale bar, 100 μm. **e** and **f** Representative vector diagrams of cell trajectories (top) and directionality of migration displayed in plot diagrams (bottom) of exosome–treated cells in each group (*n* = 20). **g** Quantification of the percentage of single cells after 12 h of migration of exosome-treated cells in each group. **h** Quantification of the persistence of cell migration of exosome-treated cells of each group (*n* = 5). Data are presented as the mean ± SD. *p < 0.05, compared with the SEs-treated group

In addition, the potential roles of serum exosomes obtained from the sham-operated and HPS model on PMVECs proliferation, migration, and tube formation were identified. Flow cytometry was applied to explore the effects of exosomes on PMVECs proliferation. The results in Fig. 2a revealed that HEs dramatically promoted PMVECs proliferation when compared to SEs-treated group (*p* < 0.05). The migration capabilities of PMVECs were detected by transwell assay. Compared with the SEs-treated group, the migration ability of PMVECs was markedly improved under the HEs treatment as displayed in Fig. 2b (*p* < 0.05). Tube formation assay was subsequently performed to confirm whether serum exosomes participated in angiogenesis. Compared with SEs, HEs obviously promoted the process of tube formation, which was revealed in Fig. 2c (*p* < 0.05). Meanwhile, our recent study reported that the directional collective migration of PMVECs was promoted in the HPS serum^36^. To explore the effect of serum exosomes on PMVECs directional collective cell migration ability, wound healing assays were utilized in the presence of SEs or HEs. Time-lapse imaging in Fig. 2d suggested that PMVECs exposed to SEs have migrated within the wounded area mainly as single cells with tortuous migration tracks, whereas cells at the edge of the wound under the HEs stimulation maintained contact with the neighboring cells and linear movement. The present assay demonstrated that Fig. 2e and f indicated a faster and directional migration of PMVECs was induced by HEs (*p* < 0.05), compared to SEs-induced PMVECs. Additionally, F-actin staining in Supplementary Fig. S2 illustrated that PMVECs stimulated by HEs up-regulated the formation of lamellipodia and filopodia compared to the SEs-treated cells.

### The miRNA expression profile of serum exosomes in HPS model

**Fig. 3:**
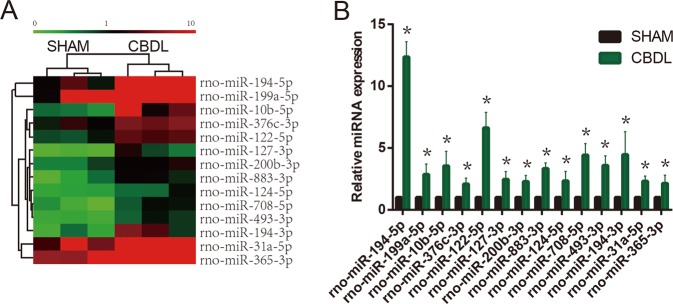
Microarray analysis reveals differentially expressed miRNAs in SEs or HEs. **a** Heat map of the 14 most highly upregulated exosomal miRNAs among 2565 miRNAs in HEs. **b** The expression profiles of the 14 most highly upregulated exosomal miRNAs were validated by qRT-PCR (*n* = 5). Data are presented as mean ± SD. *p < 0.05 compared to SEs-treated group

After being delivered into recipient cells, exosomal miRNAs are known to play important roles in various cellular functions. To characterize the serum exosome miRNome, we used microarrays to explore the miRNA spectrum present within HEs and SEs. Comparing global miRNA expression profiles in HEs with those in SEs, we identified 14 miRNAs that were significantly upregulated in HEs but not in SEs. A cluster analysis based on 14 differentially expressed microRNAs generated a tree with a clear distinction between SEs and HEs (Fig. 3a). Meanwhile, we performed qRT-PCR on 14 selected miRNAs to validate the microarray data and verified that miR-194 was the most significantly upregulated miRNA in HEs (*p* < 0.05) (Fig. 3b).

### miR-194 enhance PMVECs proliferation, migration, and tube formation

**Fig. 4:**
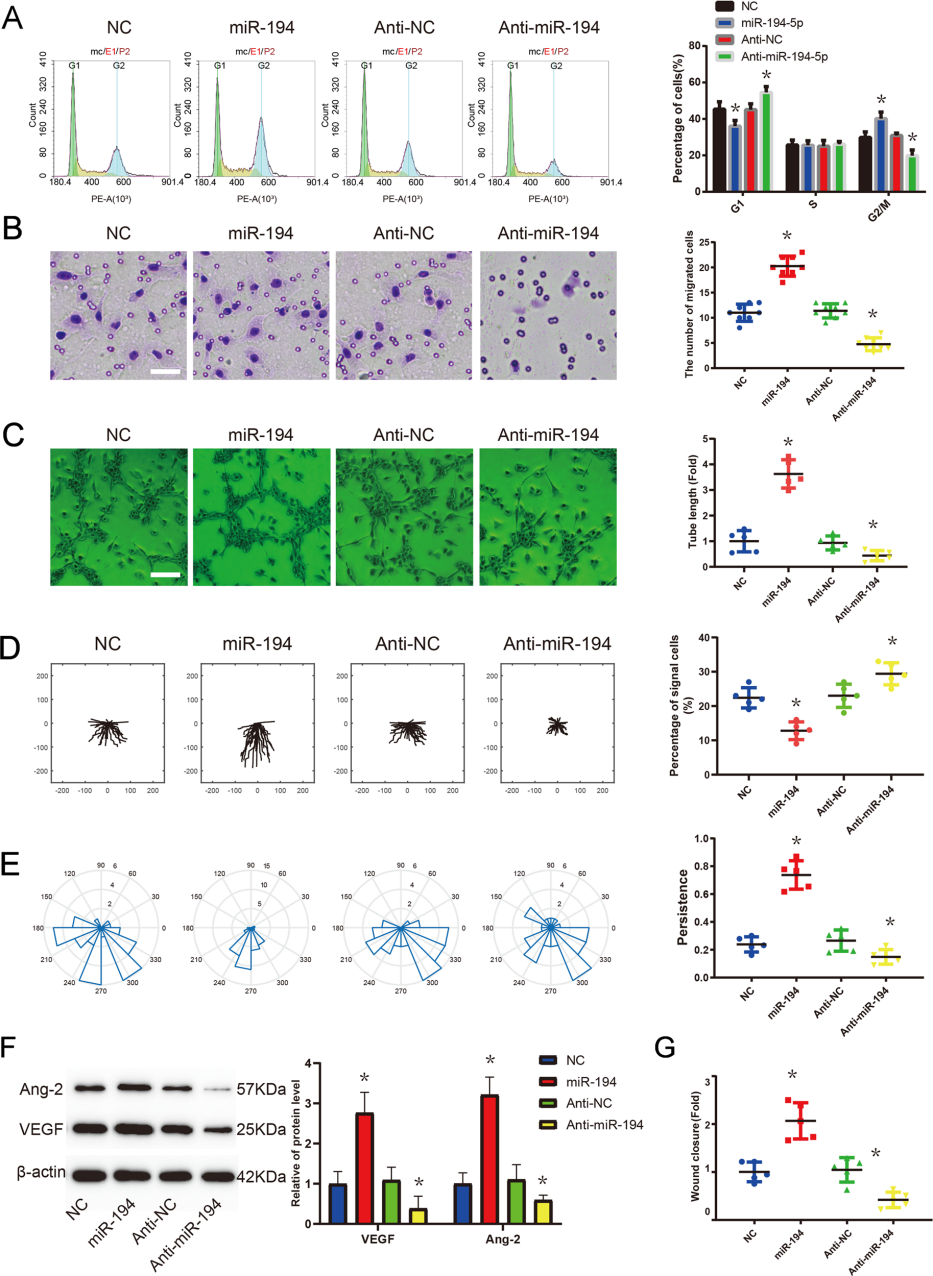
Effects of miR-194 on PMVEC proliferation, migration, and tube formation. **a**: Flow cytometric analysis was performed to detect cell cycle progression 24 h after transfection with miR-194 mimic, NC, miR-194 inhibitor, or Anti-NC (*n* = 6). **b** Transwell migration assay of PMVECs after treatment with miR-194 mimic, mimic negative control (NC), miR-194 inhibitor, or inhibitor negative control (Anti-NC) (*n* = 8). Scale bar = 50 μm. **c** Tube formation assay of PMVECs after treatment with miR-194 mimic, NC, miR-194 inhibitor, or Anti-NC (*n* = 5). Scale bar = 200 μm. **d**, **e** Representative vector diagrams of the cell trajectories (top) and directionality of migration displayed in plot diagrams (bottom) of miR-194 mimic, NC, miR-194 inhibitor, or anti-NC-treated cell in each group (*n* = 20). **g** Quantification of the percentage of single cells after 12 h of migration in miR-194 mimic, NC, miR-194 inhibitor, or anti-NC-treated cells in each group (*n* = 5). **h** Quantification of the persistence of cell migration in miR-194 mimic, mimic NC, miR-194 inhibitor, or inhibitor NC-treated cells in each group (*n* = 5). **i** Western blot analysis of Ang-2 and VEGFA expression in PMVECs after treatment with miR-194 mimic, NC, miR-194 inhibitor, or Anti-NC (*n* = 5). **j** Wound-healing assay for PMVECs after treatment with miR-194 mimic, NC, miR-194 inhibitor, or Anti-NC (*n* = 5). Data are presented as the mean ± SD. *p < 0.05, compared with NC or Anti-NC

Subsequently, the effects of miR-194 on PMVECs proliferation, migration, and tube formation were determined. The results of flow cytometry in Fig. 4a suggested that miR-194 mimics predominantly promoted PMVECs proliferation and miR-194 inhibitors significantly inhibited PMVECs proliferation when compared to the control group (*p* < 0.05). The migration capabilities of PMVECs were also investigated by transwell assay. In comparison with the control group, the migration ability of PMVECs was markedly promoted under the treatment of miR-194 mimics and dramatically suppressed treating with miR-194 inhibitors, as shown in Fig. 4b (*p* < 0.05). Tube formation assay in Fig. 4c revealed that miR-194 mimics facilitated tube formation, which was inhibited by miR-194 inhibitors (*p* < 0.05). Wound-healing assays were also adopted to explore the effect of miR-194 on PMVECs directional collective cell migration ability. Time-lapse imaging revealed that PMVECs transfected with miR-194 mimics had a faster and more directional collective migration ability, on the contrary, PMVECs transfected with miR-194 inhibitors displayed the slower and less directional collective cell migration ability (*p* < 0.05), as revealed in Fig. 4d, e, g. Moreover, Western blots results in Fig. 4f indicated that the protein levels of Ang-2 and VEGF in PMVECs at 24 h were up-regulated in the miR-194 mimics group and down-regulated in the miR-194 inhibitors group comparing with the NC mimics/inhibitors group (*p* < 0.05). Additionally, F-actin staining in Supplementary Fig. S3 suggested that PMVECs transfected with miR-194 mimics promoted and PMVECs transfected with miR-194 inhibitors inhibited the formation of lamellipodia and filopodia compared to NC mimics/inhibitors transfected cells.

### MiR-194 targeting THBS1, STAT1, and LIF may be involved in promoting pulmonary angiogenesis

**Fig. 5:**
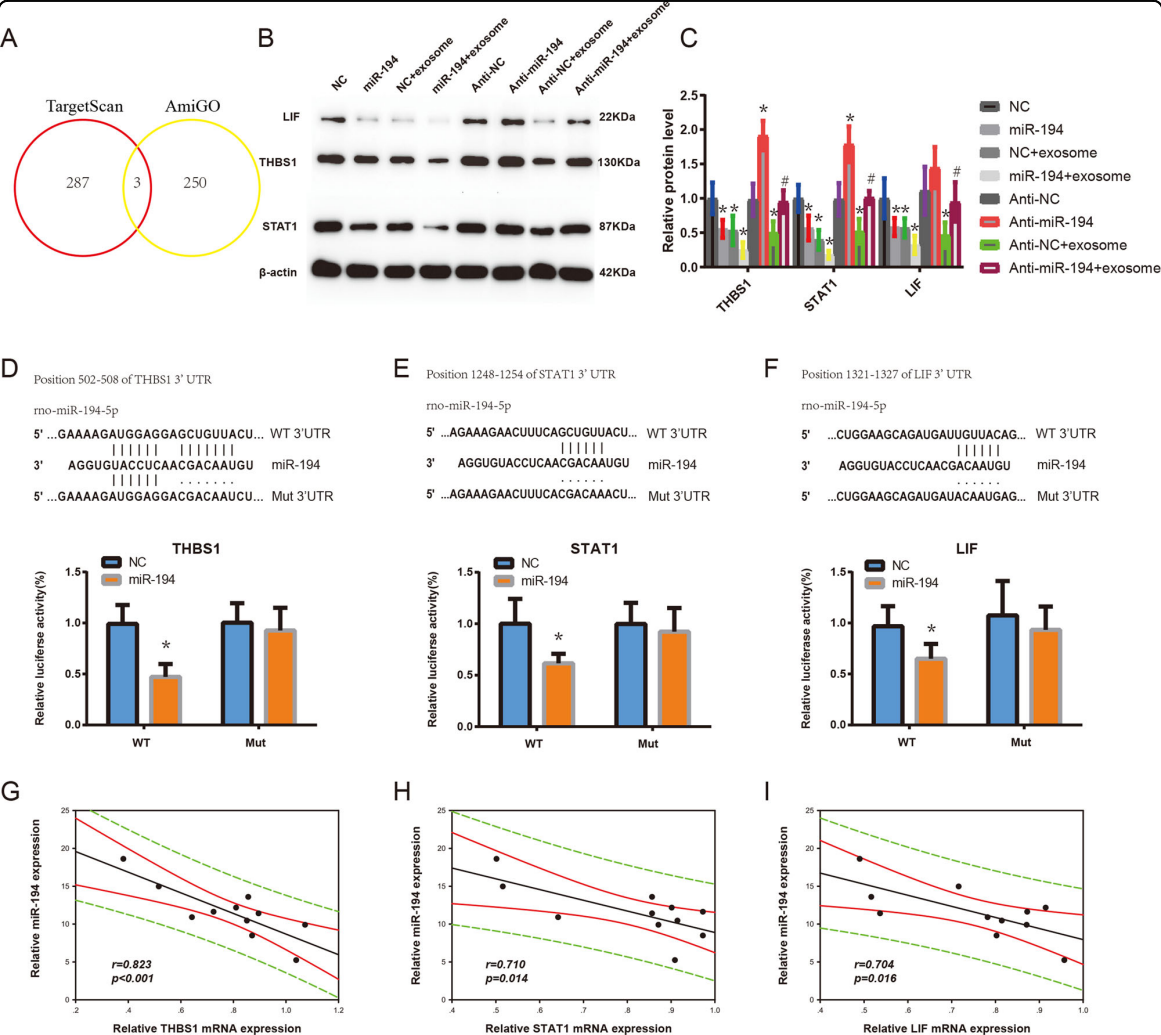
MiR-194 targeting THBS1, STAT1, and LIF may be involved in promoting angiogenesis. **a** Intersection of the TargetScan and AmiGO results. **b** Western blot analysis of THBS1, STAT1, and LIF expression in PMVECs incubated with conditioned medium, exosomes derived from HPS rats or transfected with miR-194 mimic, miR-194 inhibitor or both (*n* = 5). **c** Quantification of THBS1, STAT1, and LIF expression in PMVECs incubated with conditioned medium, exosomes derived from HPS rats or transfected with miR-194 mimic, miR-194 inhibitor, or both. **d**–**f** miR-194 inhibited the luciferase activity of reporter containing the wild-type but not mutant 3′-UTR of THBS1, STAT1, and LIF. Upper panel, miR-194 and its putative binding sequence in the 3′-UTR of THBS1, STAT1, and LIF. Mutation sequences are underlined. Lower panel, cells were co-transfected with NC or miR-194 mimics, pRL-TK and a firefly luciferase reporter plasmid carrying the wild-type (WT) or mutant (MUT) 3′-UTR. The luciferase activity of NC-transfectants from one of the experiments in the WT group was set as 1. pRL-TK that expressed Renilla luciferase was used to correct the difference in transfection and harvest efficiencies (*n* = 5). Data are presented as the mean ± SD. *p < 0.05, compared with NC control group; ^#^*p* < 0.05, compared with anti-NC + exosome group. **g**–**i** Correlation of serum exosomal miR-194 and the expression of THBS1, STAT1, and LIF in PMVECs isolated from HPS rats (*n* = 20)

We next explored how the secreted miR-194-induced angiogenesis. Putative targets of miR-194 were predicted based on TargetScan databases (http://www.targetscan.org/). Genes, which negatively regulate angiogenesis, were collected from AmiGO (http://amigo.geneontology.org/). The overlap genes between the full miR-194-target gene lists of TargetScan and negatively regulated angiogenesis gene lists are THBS1, STAT1, and LIF (Fig. 5a and Supplementary Tables 1 and 2).

Western blotting results demonstrated that PMVECs treated with HEs or miR-194 mimics exhibited a remarkable reduction in THBS1, STAT1, and LIF expressions. Furthermore, PMVECs treated with HEs and miR-194 mimics showed a further suppression in the above expressions. However, THBS1, STAT1, and LIF expressions were up-regulated after treating with the miR-194 inhibitor. Moreover, the transfection of miR-194 inhibitor into PMVECs significantly abolished the capacity of HEs to decrease the THBS1, STAT1, and LIF expressions in PMVECs (Fig. 5b, c). The above results implied that serum exosomal-miR-194 may promote angiogenesis of PMVECs by inhibiting the expression of anti-angiogenesis molecules.

Eventually, the dual-luciferase reporter assay suggested that co-transfection of miR-194 significantly inhibited the activity of firefly luciferase reporter carrying wild-type 3′-UTR of THBS1, STAT1, or LIF (*p* < 0.05), whereas this effect was abrogated when the predicted binding site in 3′-UTR was mutated (*p* > 0.05) (Fig. 5d–f). we further evaluated the transcription expression of these three target genes in PMVECs isolated from HPS rats, and statistically significant inverse correlations were observed for miR-194-THBS1, miR-194-STAT1, and miR-194-LIF (*r* = −0.823, *p* = 0.001; *r* = −0.710, *p* = 0.014; *r* = −0.704, *p* = 0.016 (Fig. 5g, h, i). It can be illustrated from the results that serum exosomal miR-194 obtained from HPS rats may be involved in promoting pulmonary angiogenesis by directly targeting THBS1, STAT1, and LIF.

### Bile acid overload induced p53 nuclear translocation promotes the production of exosomes from hepatocyte

**Fig. 6:**
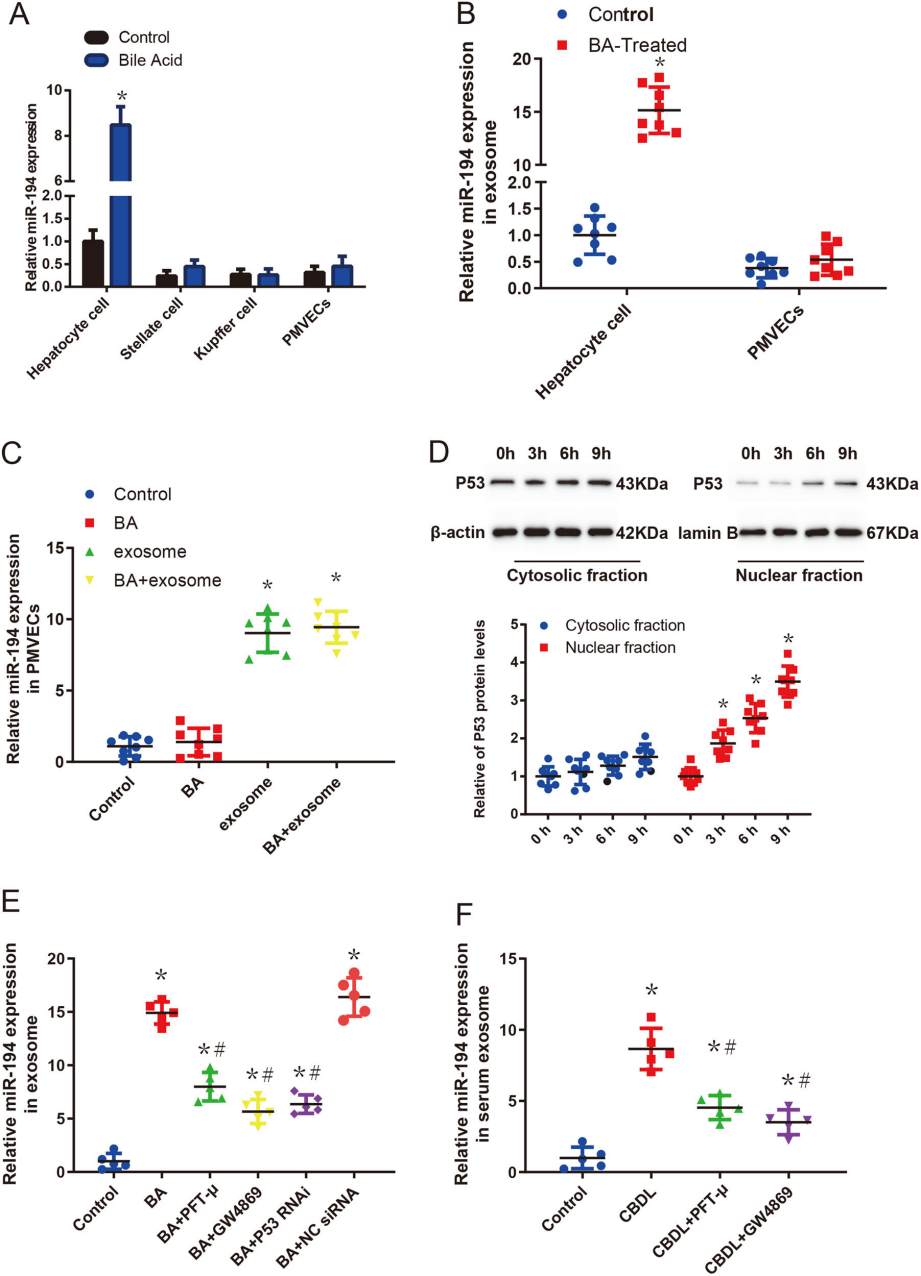
Bile acid overload-induced p53 nuclear translocation promotes the production of exosomes from hepatocytes. **a** Quantitative real-time PCR of mature miR-194 in cultured rat Kupffer cells, stellate cells, hepatocytes, and PMVECs at 24 h after treatment with bile acid (GCDC, 50 μmol) (*n* = 5). Data are presented as the mean ± SD. *p < 0.05, compared with the control group. **b** Levels of miR-194 expression in exosomes from in vitro cultured rat primary hepatocytes and PMVECs cell culture medium at 24 h after treatment with bile acid (GCDC, 50 μmol) (*n* = 8). Data are represented as mean ± SD. *p < 0.05, compared with the control group. **c** Levels of miR-194 expression in PMVECs at 24 h after treatment with bile acid or hepatocyte-derived exosomes or both (*n* = 8). Data are presented as the mean ± SD. *p < 0.05, compared with the control group. **d** p53 was upregulated and translocated to the nucleus after bile acid treatment. Endogenous p53 in both the cytoplasm (non-NE) and nucleus (NE) of hepatocytes, which were treated with bile acid for different time point, was detected using anti-p53, β-actin, and lamin B antibodies for Western blotting (*n* = 8). Data are presented as the mean ± SD. *p < 0.05, compared with 0 h. **e** Levels of miR-194 expression in exosomes from in vitro cultured rat primary hepatocytes after which were treated with bile acid and 10 μM GW4869, 10 μM pifithrin-μ, P53 RNAi, NC RNAi for 24 h (*n* = 5). *p < 0.05, compared with the control group. ^#^*p* < 0.05 compared with the BA group. **f** Levels of miR-194 expression in serum exosomes in the sham, 5-week CBDL rats with or without pifithrin-μ, or GW4869 administration. Data are presented as the mean ± SD (*n* = 5). *p < 0.05, compared with the sham-operated group; ^#^*p* < 0.05 compared with CBDL group

The cells secreted miR-194 in HEs were also examined. The previous study indicated that miR-194 was highly expressed in hepatocytes, exhibiting a similar expression pattern with the liver-specific miRNA^37^. In our study, the miR-194 expressions in hepatocytes, Kupffer cells, stellate cells, and PMVECs were evaluated and it was shown in Fig. 6a, where it was highly expressed in hepatocytes, and further increased in bile acid treated hepatocytes (*p* < 0.05). Therefore, we hypothesized that bile acid-induced hepatocyte-derived exosomal miR-194 might up-regulate the miR-194 expression in PMVECs. To verify this hypothesis, bile acid was adopted to stimulate hepatocytes and PMVECs separately, then the exosomal miR-194 expression was analyzed. As shown in Fig. 6b and Supplementary Fig. S3B, compared with the control group, both the expressions of miR-194 and exosomal miR-194 were significantly increased in the hepatocyte culture medium and hepatocyte cells within 24 h of the bile acid treatment (*p* < 0.05). Meanwhile, there was a 5.2-fold increase of the exosome concentration in the hepatocyte culture medium within 24 h of the bile acid treatment as represented in Supplementary Fig. S4A (*p* < 0.05). However, Fig. 6b, c demonstrated that the expressions of miR-194 and exosomal miR-194 were only slightly increased in PMVEC culture medium and PMVECs after the bile acid administration (*p* > 0.05).

Besides, the bile acid-induced hepatocyte-derived exosomes were adopted to culture with PMVECs, which was indicated in Fig. 6c that the miR-194 expression in PMVECs were dramatically boosted (*p* < 0.05). The above result indicated that hepatocyte-derived exosomes can directly up-regulate miR-194 expression in PMVECs. However, it was still unknown which signaling pathway mediated the bile acid-induced increase of exosome concentration and miR-194 levels. According to our former study and other related research, P53 were reported markedly up-regulated in the nucleus in CBDL-stimulated rat model^38–40^. Herein, it was illustrated in Fig. 6d that bile acid facilitated the accumulation of P53 in nucleus in a time-dependent manner (*p* < 0.05). In addition, the exosome biogenesis related to tumor suppressor activated pathway 6 (TASP6) and miR-194 expression could be promoted by the P53 gene^41–47^. Therefore, P53 inhibitor pifithrin-μ, P53 RNAi was utilized to explore the effect of P53 on TASP6 expression, exosome and exosomal miR-194 secretion in the culture medium after treating with bile acid. It was revealed in Fig. 6e, Supplementary Fig. S3A and D that both pifithrin-μ and P53 RNAi markedly inhibited the TASP6 expression, exosome and exosomal miR-194 secretion (*p* < 0.05). The effect of the SMase inhibitor GW4869 which is the general inhibitor of EV secretion on exosome and exosomal miR-194 secretion was also investigated. The results were displayed in Fig. 6e and Supplementary Fig. S4A show that the exosome and exosomal miR-194 secretion were dramatically decreased by GW4869 (*p* < 0.05). Additionally, pifithrin-μ and GW4869 were injected i.v. to evaluate their effects on the levels of serum exosome and exosomal miR-194 in CBDL-treated rats. Figure 6f and Supplementary Fig. S4C revealed that pifithrin-μ and GW4869 predominantly reduced the levels of serum exosome and exosomal miR-194 (*p* < 0.05).

### Serum exosomal miR-194 modulates pulmonary angiogenesis in CBDL-induced rats

**Fig. 7:**
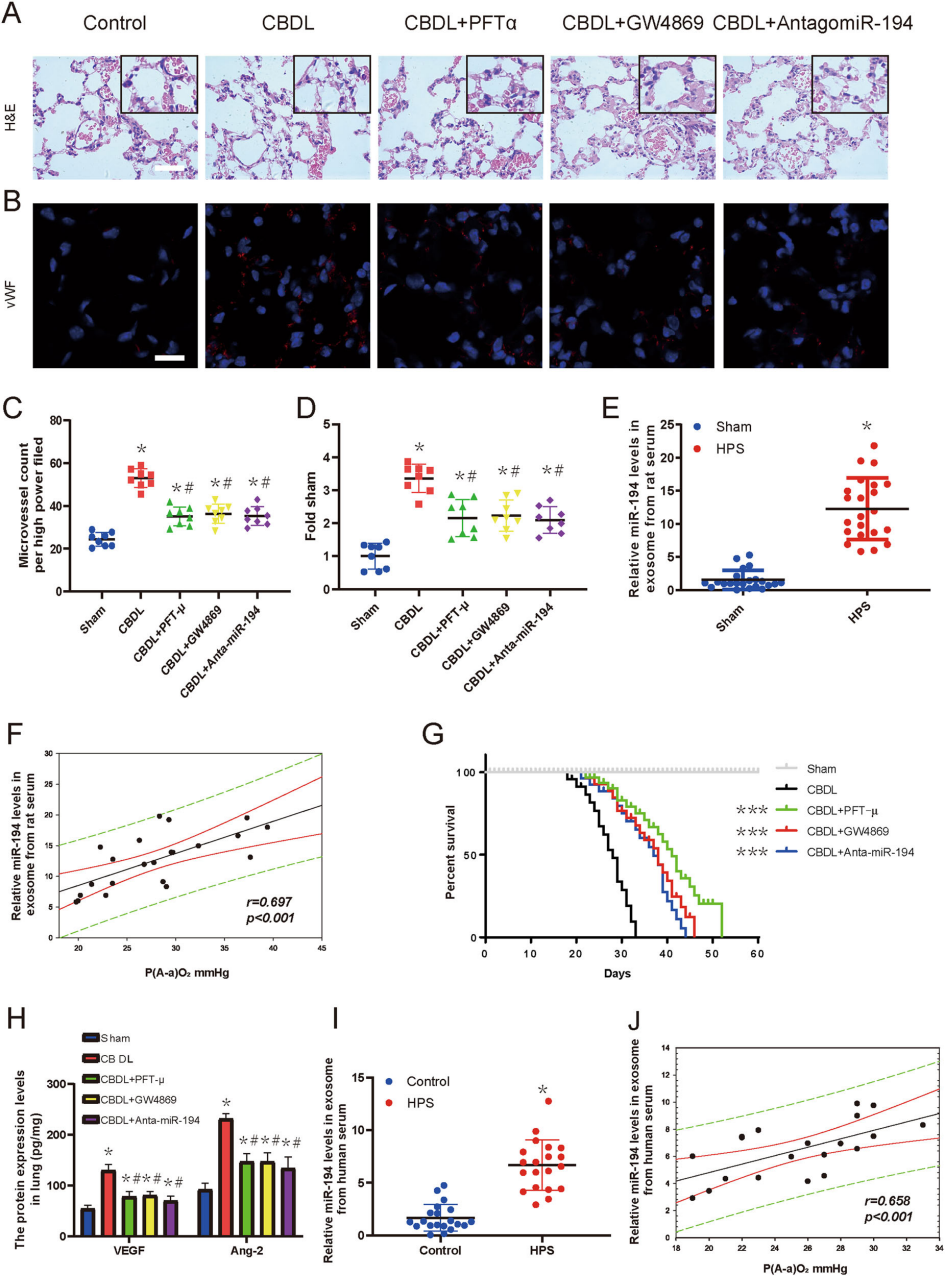
Serum exosomal miR-194 modulates pulmonary angiogenesis in CBDL rats. **a**, **c** Representative micrographs of hematoxylin and eosin staining of pulmonary microvessels in sham-operated, 5-week-old CBDL rats with or without pifithrin-μ, GW4869, or anti-miR-194 administration, and graphical summaries of the microvessel count in sham, 5-week-old CBDL rats with or without pifithrin-μ, GW4869, or anti-miR-194 administration. Scale bar, 100 μm. **b**, **d** Immunostaining of vWF (red) with DAPI nuclear staining (blue), and graphical summaries of the microvessel count in the sham-operated, 5-week-old CBDL rats with or without pifithrin-μ, GW4869, or anta-miR-194 administration (*n* = 8). Scale bar, 250 μm. Data are presented as the mean ± SD. *p < 0.05 compared with the sham group. ^#^*p* < 0.05 compared with the CBDL group. **e** qRT-PCR analysis was performed to detect the expression of serum exosomal miR-194 in the HPS group and the sham-operated group (*n* = 22). Data are represented as mean ± SD. *p < 0.05 compared with the sham group. **f** Correlation between exosomal miR-194 and P(A-a)O_2_ in HPS rats (*n* = 22) were compared using a Pearson correlation test. **g** Kaplan–Meier plot of overall survival. The differences between survival curves for 60 days in groups (Sham group, *n* = 10; CBDL group, *n* = 21; CBDL + pifithrin-μ group, *n* = 20; CBDL + GW4869 group, *n* = 20; CBDL + anta-miR-194 group, *n* = 20) were compared using a log-rank test. ***p < 0.005 versus CBDL. **h** ELISA analysis of the Ang-2 and VEGF protein expression levels in lung tissue after treatment with SEs or HEs (*n* = 5). Data are represented as mean ± SD. *p < 0.05 compared with the sham-operated group. **i** qRT-PCR analysis was performed to detect the expression of serum exosomal miR-194 in HPS patients and control group (*n* = 20). Data are represented as mean ± SD. *p < 0.05 compared with the control group. **j** Correlation between exosomal miR-194 and P(A-a)O_2_ in HPS patients (*n* = 20) were compared using a Pearson correlation test

To explore whether altered serum exosomal miR-194 modulated pulmonary angiogenesis, CBDL-induced rats were injected i.v. with pifithrin-μ, GW4869, or anta-miR-194. Angiogenesis was assessed by quantifying microvessels and measuring vWF levels as published previously^48^. Compared with control animals, where angiogenesis in the pulmonary microvasculature was observed 5 weeks after the CBDL administration, reflected by a marked increase in microvessel counts and vWF levels (*p* < 0.05). Pifithrin-μ, GW4869, or anta-miR-194 administration in CBDL animals resulted in a conspicuous reduction in pulmonary microvessel counts and lung vWF levels, indicating a remarkable inhibition of angiogenesis (*p* < 0.05), as revealed in Fig. 7a–d.

To compare the expression abundance of exosomal miR-194 in HPS and sham-operated controls, qRT-PCR analysis was performed with exosomes isolated from CBDL and sham-operated rats. As shown in Fig. 7e, compared with the sham-operated group, the expression of exosomal miR-194 was dramatically up-regulated in HPS rats serum (*p* < 0.05). Furthermore, the correlation between the expression of serum exosomal miR-194 and P(A-a)O_2_ in HPS rats were further analyzed by the Pearson correlation test. As presented in Fig. 7f, significant positive correlation between the level of miR-194 and P(A-a)O_2_ were observed in HPS rats (*p* < 0.05). We further verified that exosomal miR-194 was significantly increased and positively correlated with P(A-a)O_2_ in HPS patients serum (*p* < 0.05) (Fig. 7i, j). Next, the effects of pifithrin-μ, GW4869, and anta-miR-194 on the survival of CBDL rat were evaluated. It was shown in Fig. 7g that the survival rates of the pifithrin-μ, GW4869, and anta-miR-194-treated CBDL group were higher than the non-treated CBDL groups (*p* < 0.005). Additionally, ELISA results showed that the protein levels of Ang-2 and VEGF in lung tissue homogenates were significantly increased in CBDL group (*p* < 0.05). The groups treated with pifithrin-μ, GW4869, or anta-miR-194 showed a marked reduction in Ang-2 and VEGF levels compared with the CBDL group (*p* < 0.05) (Fig. 4h). Taken together, these results showed that serum exosomal miR-194 modulates pulmonary angiogenesis and overall survival of rats with CBDL.

## Discussion

**Fig. 8:**
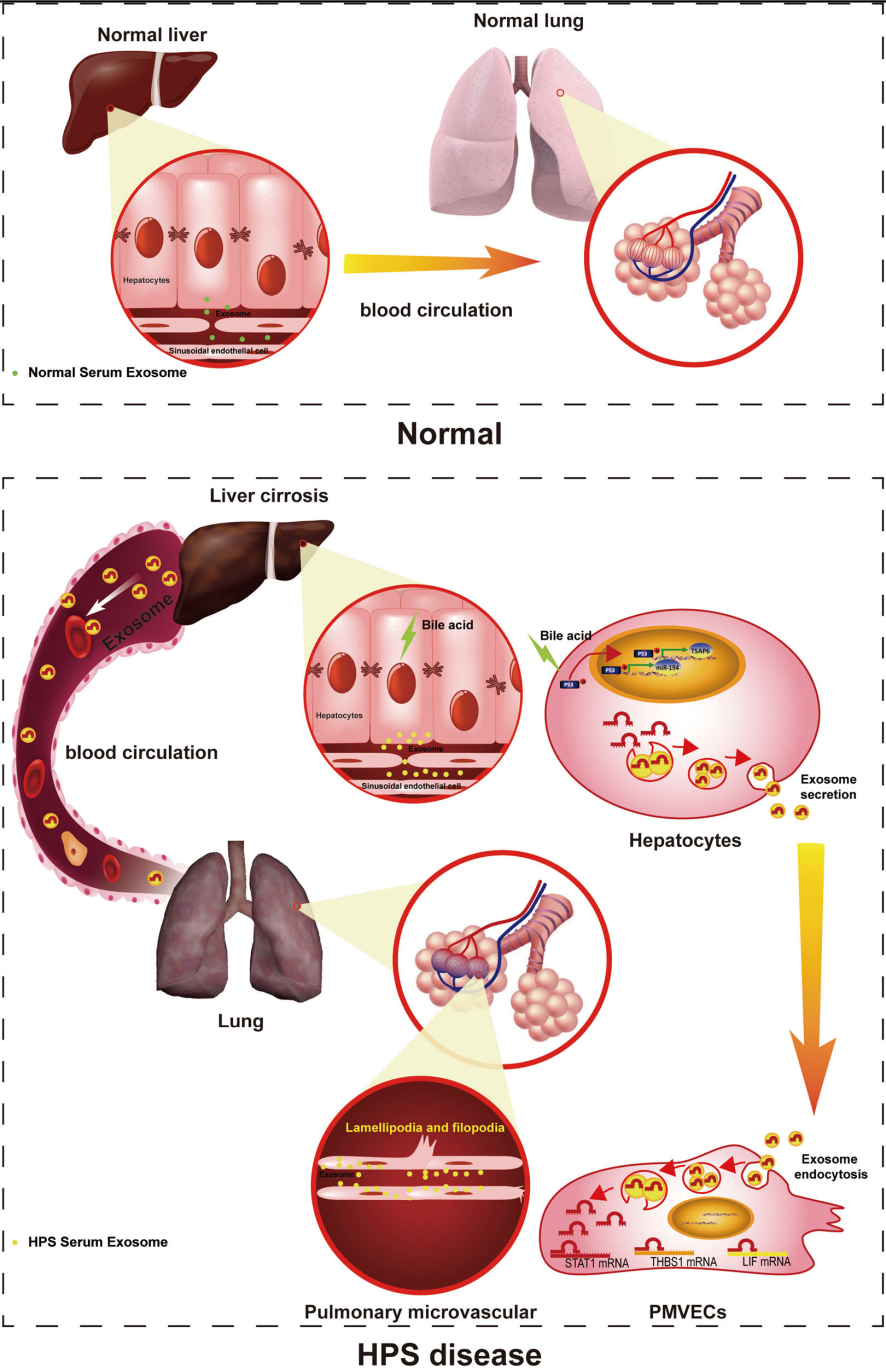
Schematic showing the working model of our study. Hepatocytes secrete exosomes containing miR-194 upon stimulation with bile acid through the P53-signaling pathway, which are internalized by PMVECs. Additionally, exosomal miR-194 enhances PMVEC proliferation, migration, and tube formation by directly targeting THBS1, STAT1, and LIF. The communication between hepatocytes and PMVECs via exosomal miR-194 augments the angiogenesis signal from hepatocytes and promotes the development of HPS

The pathophysiological mechanisms associated with HPS are complicated and remain incompletely identified^1,2,49^. Recently, an increasing number of studies have identified that angiogenesis caused by angiogenic factors also plays an important role in HPS^50,51^. In this study, we identified that HEs significantly increased PMVEC proliferation, migration, and tube formation. We performed an exosomal miRNA analysis using microarray and identified the expression of miR-194 that was most significantly increased in HEs. Furthermore, we demonstrate a novel mechanism through which the exosome/miR-194 axis mediated cellular communication between hepatocytes and PMVECs and subsequently induced pulmonary angiogenesis (Fig. 8). Our findings may provide a novel molecular target for the treatment of HPS.

Angiogenesis is the process by which new capillary formation occurs from the preexisting vasculature. This process is determined by endothelial cell proliferation and migration, and depends on the balance of proangiogenic and antiangiogenic factors^52,53^. Zhang et al. found that pulmonary angiogenesis occurs selectively in CBDL animals and that pentoxifylline directly decreases the number of microvessels, downregulates pulmonary angiogenic factors, and reduces the symptoms of HPS^54^. Chang et al. found that rosuvastatin alleviates experimental HPS through blockade of pulmonary inflammatory angiogenesis via downregulation of the TNF-α/NF-κB and VEGF/Rho-associated A kinase pathways^10^. Our previous study found that Caspase-3 inhibition alleviates angiogenesis as well as the development of HPS in CBDL rats. These effects are related to blocking the induction of a rescue angiogenic program^48^. However, further study is needed to identify the mechanism of the specific hepatic factors that contribute to angiogenesis after CBDL.

Recent studies have indicated that angiogenesis can also be modulated by distant cell-derived exosomes^55,56^. For instance, Tang et al. found that sE-cad-containing exosomes were present and able to induce angiogenesis in HCT116 colon cancer and MCF-7 breast cells. Moreover, sE-cad-positive exosomes can also be widely found in ascitic fluids of different patients with different types of cancers, suggesting that the angiogenic function of sE-cad-containing exosomes may have broader implications^57^. Zeng et al. observed that colorectal cancer cell-derived exosomal miR-25-3p promotes premetastatic niche formation by inducing vascular permeability and angiogenesis^58^. In this study, for the first time, we observed that exosomes collected from CBDL rat serum entered endothelial cells, resulting in enhanced proliferation, migration, and tube formation.

Meanwhile, accumulating evidence from the literature supports the idea that exosomal miRNAs can act as regulators of angiogenesis in distant cells. For example, it has been shown that CD105 cancer stem cell-derived miRNA-enriched exosomes can modify the tumor microenvironment by triggering angiogenesis^59^. The miR-17-92 cluster, as a specific exosomal miRNA, plays an important role in regulating endothelial gene expression during tumor angiogenesis in leukemia cells^60^. Moreover, exosomal angiogenic miR-210, which is increased in the serum of malignant breast cancer patients, regulates the process of tumor angiogenesis by suppressing specific target genes^61^. To figure out the functional contents in exosomes, we conducted a microarray analysis to investigate the different miRNA profiles in these two kinds of exosomes. As a result, miR-194 was significantly upregulated in CBDL rat serum-derived exosomes and that miR-194 promoted the proliferation, migration, and tube formation of PMVECs. Because miRNAs can act either as tumor suppressors or oncogenes, depending on their target genes in different recipient cells, we identified three anti-angiogenesis genes (THBS1, STAT1, LIF) as target gene of miR-194. We further observed that inverse correlations for miR-194-THBS1, miR-194- STAT1, and miR-194- LIF in PMVECs isolated from CBDL rats.

P53, which is known as the ‘guardian of the genome’ or ‘cellular gatekeeper,’ is a crucial gene in multicellular organisms^62^. As a transcription factor, p53 functions to control cell fate under various types and levels of cellular stress through its downstream target genes^63^. Although best known for its canonical functions of inducing DNA repair, cell cycle arrest, and apoptosis, p53 has also been revealed to govern the release of exosomes from cells^64^. DNA damage or other cellular stress-induced p53 activation can up-regulate the expression of TSAP6, which has been found to be essential for p53-mediated exosome release^65^. Additionally, p53 also upregulates specific miRNA expression and transfers miRNA into exosomes^45,47^. As a pro-angiogenic miRNAs, p53-responsive miR-194 has been reported to mediate astroglial–endothelial cellular transition and regulates expression of THBS1, an anti-angiogenic factor compromising endothelial cell survival, migration, and responses to the VEGF. Although the high expression of miR-194 in the hepatocytes has been known for a long time, its function is poorly understood. Through this study, we found that exosomal miR-194 secretion was inhibited by GW4869, a general inhibitor of EV secretion that acts by blocking the classical EV biogenesis pathway, pifithrin-μ, an inhibitor of P53, or P53 RNAi both in vivo and in vitro. Moreover, we also demonstrated pifithrin-μ, GW4869, or anta-miR-194 can inhibit CBDL-induced pulmonary angiogenesis. Therefore, regulation of exosomal miR-194 secretion may play an important role in HPS progression.

Exosomes stemming from body fluids, such as serum, plasma, breast milk, saliva, urine, amniotic fluid, and cerebrospinal fluid, contain substantial amounts of miRNAs^66,67^. Recently, serum exosomal miRNA profiles of different diseases were carried out. They reach recipient cells by passing through these body fluids, fulfilling their functions as signal vehicles in physiology and pathology^68,69^. In the present study, we found that hepatocytes-derived serum exosomal miR-194 play an important role in HPS. However, there remain some limitations that we should acknowledge. First of all, the sample size enrolled in this study for microarray was relatively small, which might neglect other functional miRNAs in the pathological processes of HPS. Secondly, due to the complicated exosomal contents, the exosome/miR-194 axis pathway might only be one of multiple pathways that regulate angiogenesis. The residual proteins in the purified exosomes might also play a role in exosome regulation.

In conclusion, we demonstrated that exosomal miR-194 mediates the cross-talk between hepatocytes and PMVECs and contributes to PMVEC proliferation, migration, and tube formation. The exosome/miR-194 axis plays a critical pathologic role in pulmonary angiogenesis. The findings suggest that exosomal miR-194 may represent a new therapeutic target for inhibiting the progression of HPS.

## Supplementary information

Supplementary Figure Legends

Supplementary Figure 1

Supplementary Figure 2

Supplementary Figure 3

Supplementary Figure 4

Supplementary Figure 5

Negative regulation of angiogenesis-related gene list

Target gene list of miR-194

Up-regulated miRNAs detected by miRCURY LNA Arrays (Exiqon) in HEs and SEs

Contribution form
