# Supplementary material for: Hepatocyte-derived exosomal MiR-194 activates PMVECs and promotes angiogenesis in hepatopulmonary syndrome
Source: Cell Death Dis. 2019 Nov 7;10(11):853. doi: 10.1038/s41419-019-2087-y (PMC6838168; doi:10.1038/s41419-019-2087-y)
Supplement: Supplementary file 1 — Supplementary Figure Legends [file 41419_2019_2087_MOESM1_ESM.docx]

**Supplementary Figure 1. Incorporation of hepatic exosomes into lung tissue.** CBDL rats were administered PKH67-labeled SEs or HEs from via tail vein injection. Lung were harvested and cutted into 5-um thickness sections at 3h after injection and imaged using a fluorescent microscope (Olympus BX51, Tokyo, Japan). Two representative confocal images of each condition are depicted. PKH67-label is in green, VWF-label is in red and DAPI (nucleus) is in blue. Scale bar: 250 μm.

**Supplementary Figure 2. HEs induces lamellipodia and filopodia in cultured PMVECs**. After incubation with SEs or HEs for 24h, PMVECs were stained with Alexa Fluor 546-coupled phalloidin to visualize F-actin. Two representative confocal images of each condition are depicted. Phalloidin-label is in red and DAPI (nucleus) is in blue. Scale bar: 10 μm.

**Supplementary Figure 3. miR-194 induces lamellipodia and filopodia in cultured PMVECs**. After transfection with miR-194 mimic, mimic NC, miR-194 inhibitor, or inhibitor NC, PMVECs were stained with Alexa Fluor 546-coupled phalloidin to visualize F-actin. Representative confocal images of each condition are depicted. Phalloidin-label is in red and DAPI (nucleus) is in blue. Scale bar: 10 μm.

**Supplementary Figure 4. Bile acid overload induced p53 nuclear translocation promote the production of exosomes from hepatocyte.** A: Relative exosome concentration in hepatocyte cell culture media after hepatocyte cells were treated with bile acid and 10μM GW4869 or 10μM pifithrin-μ or P53 RNAi or NC RNAi for 24 hour (n = 8). Data are presented as the mean ± SD. ^*^P<0.05, compared with control group. ^#^P<0.05 compared with BA group. B: Relative concentration of exosome derived from rat peripheral blood serum in study groups (n = 5). Data are presented as the mean ± SD. ^*^P<0.05, compared with sham group; ^#^P<0.05 compared with CBDL group. C: Levels of miR‐194 expression in exosomes and cell lysates from in vitro cultured rat primary hepatocytes after which were treated with bile acid and 10μM GW4869 or 10μM pifithrin-μ or P53 RNAi or NC RNAi for 24 hour (n = 8). Data are presented as the mean ± SD. *P<0.05, compared with control group. ^#^P<0.05 compared with BA group. D：qRT-PCR analysis of TSAP6 expression in hepatocytes incubated with conditioned medium, PBS or pifithrin-μ or GW4869 or transfected with P53 RNAi or NC RNAi for 24 h (n = 5). Data are presented as the mean ± SD. ^*^P<0.05, compared with control group; ^#^P<0.05 compared with BA group.

**Supplementary Figure 5. Hepatocyte derived exosome induced by bile acid can be absorbed by PMVECs and serum exosomal miR-194 may mainly come from hepatocyte derived exosomes**

A: The detection of exosome uptake by PMVECs in vitro. Exosomes are shown (PKH67 in green, DAPI in blue). Scale bar, 50 μm. B: Expression of miR-194 in the liver or lung exosomes from CBDL rats. Data are presented as the mean ± SD. ^*^P<0.05, compared with liver exosomes group. C,D: ELISA analysis of the ALB and HB protein expression levels in CEs or HEs (n = 11). Data are represented as mean ± SD. ^*^P < 0.05 compared with the sham-operated group. E, F: Correlation between exosomal miR-194 and hepatocyte-specific proteins in HEs (n = 11) were compared using a Pearson correlation test.
