# Supplementary figures and images for: Hepatocyte-derived exosomal MiR-194 activates PMVECs and promotes angiogenesis in hepatopulmonary syndrome
Source: Cell Death Dis. 2019 Nov 7;10(11):853. doi: 10.1038/s41419-019-2087-y (PMC6838168; doi:10.1038/s41419-019-2087-y)

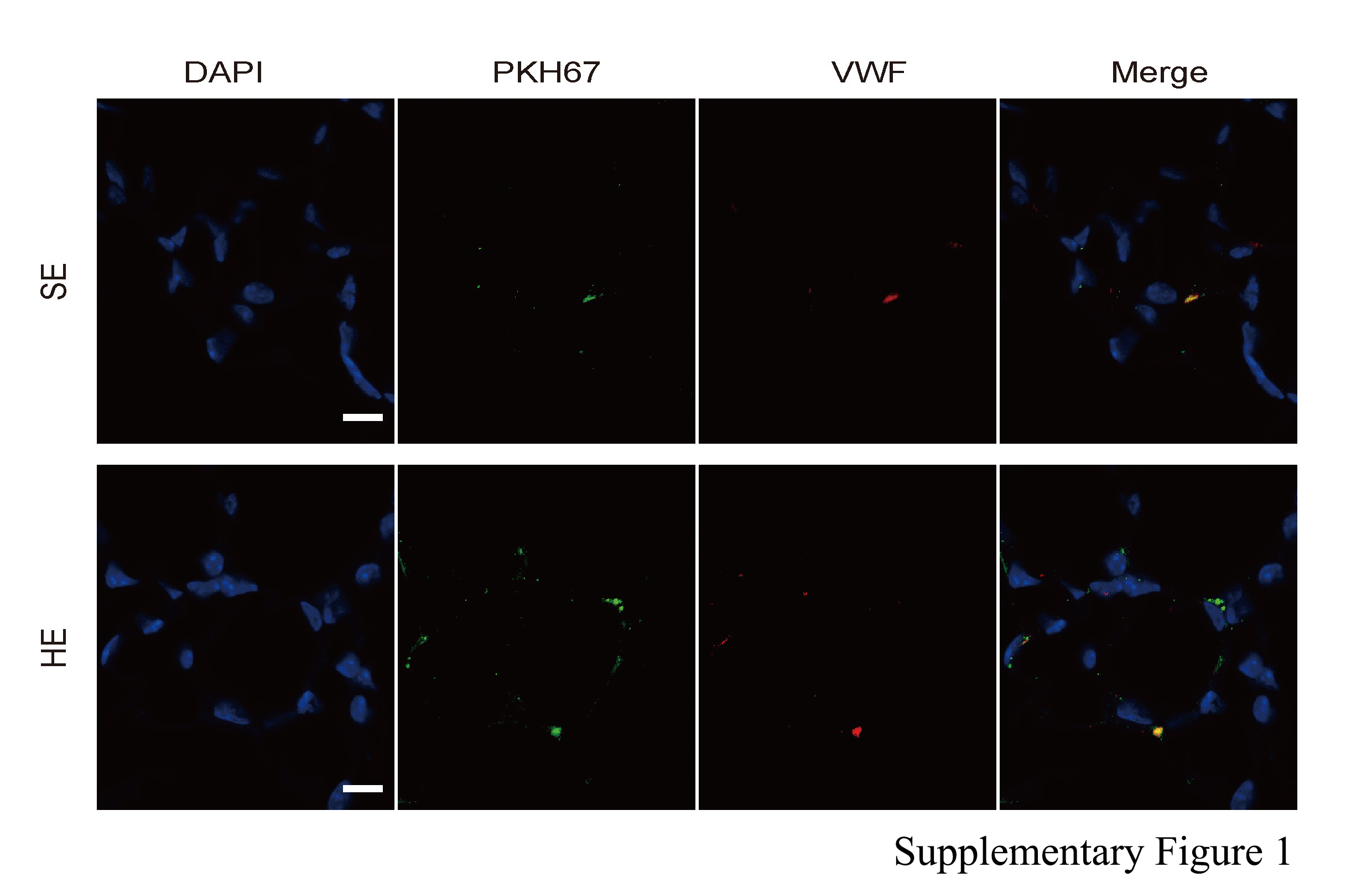

Supplement: Supplementary file 2 — Supplementary Figure 1 [file 41419_2019_2087_MOESM2_ESM.tif]

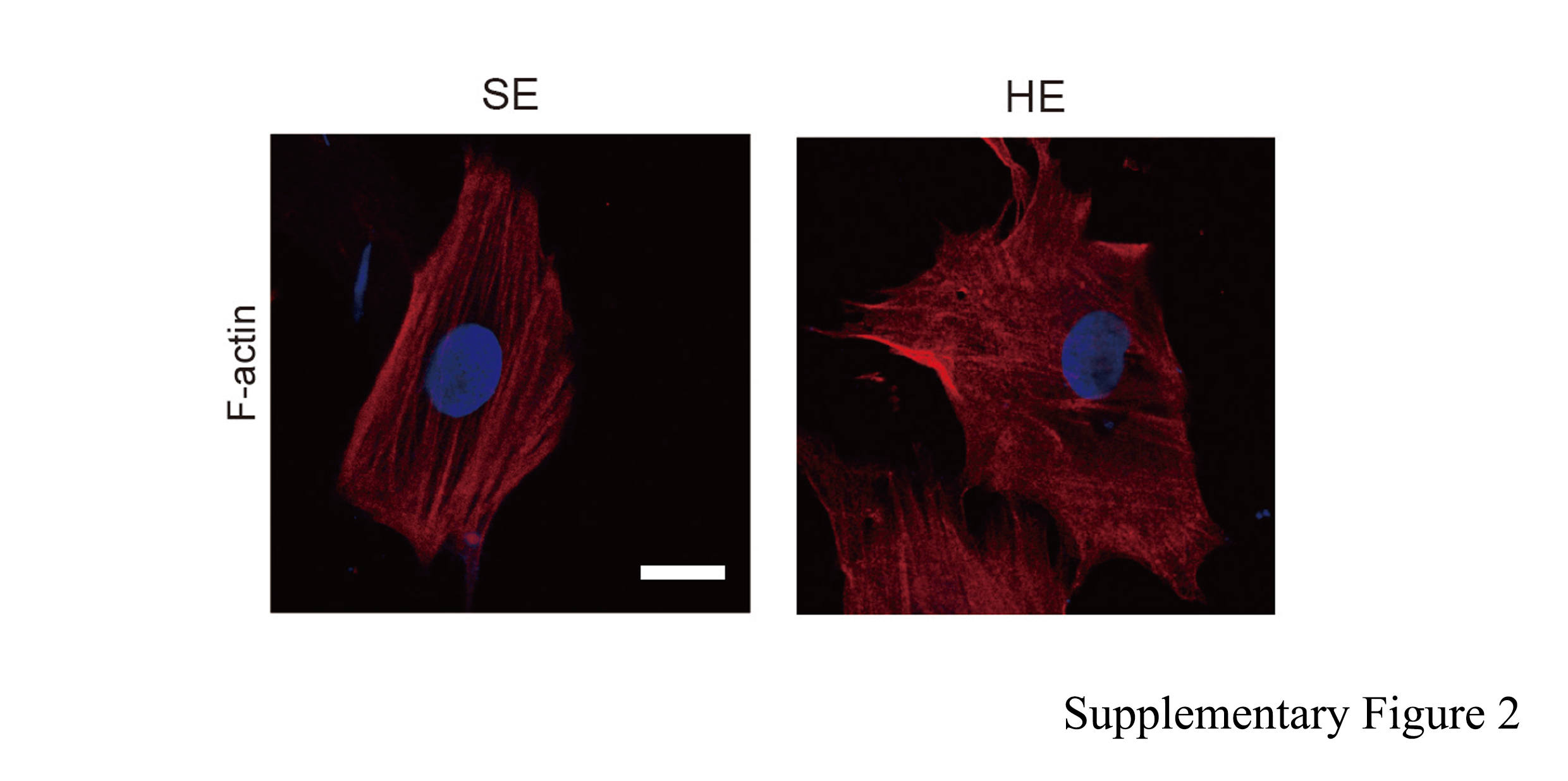

Supplement: Supplementary file 3 — Supplementary Figure 2 [file 41419_2019_2087_MOESM3_ESM.tif]

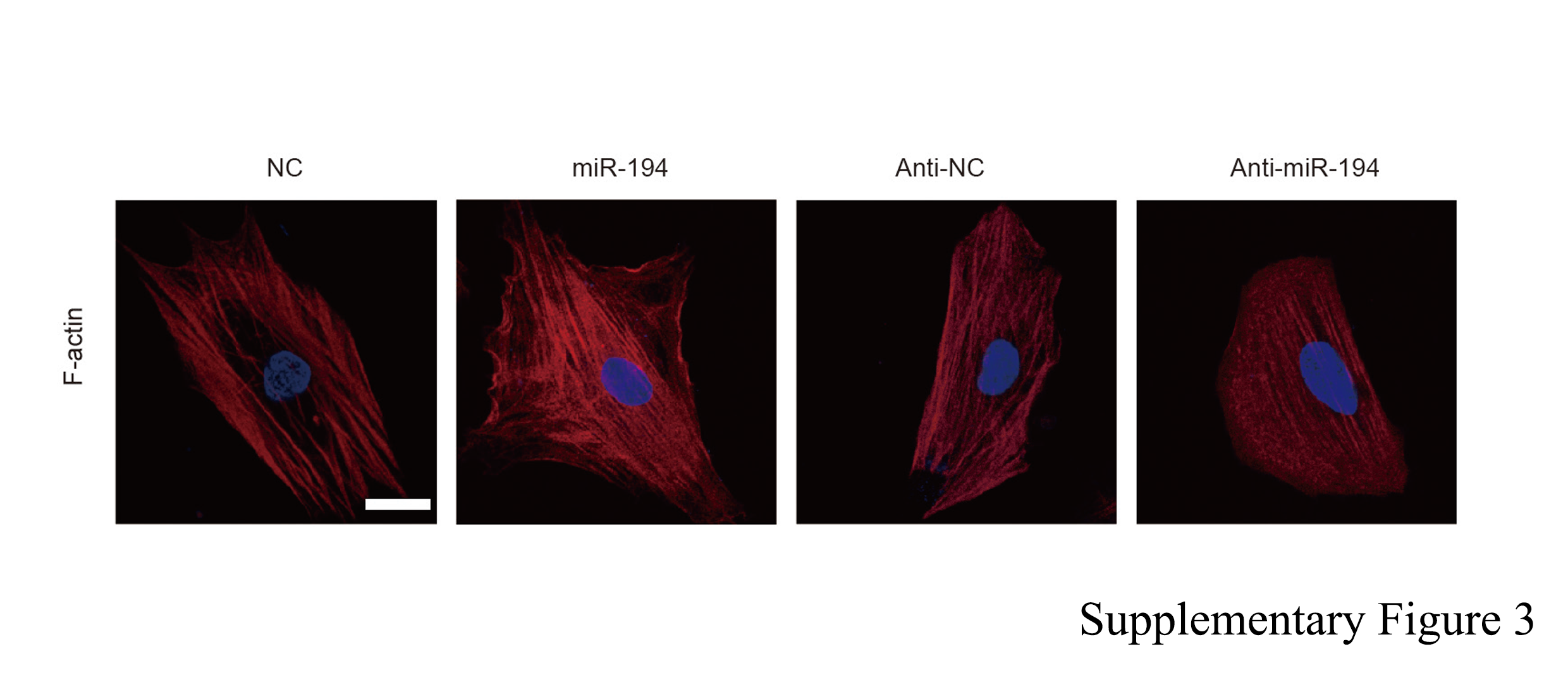

Supplement: Supplementary file 4 — Supplementary Figure 3 [file 41419_2019_2087_MOESM4_ESM.tif]

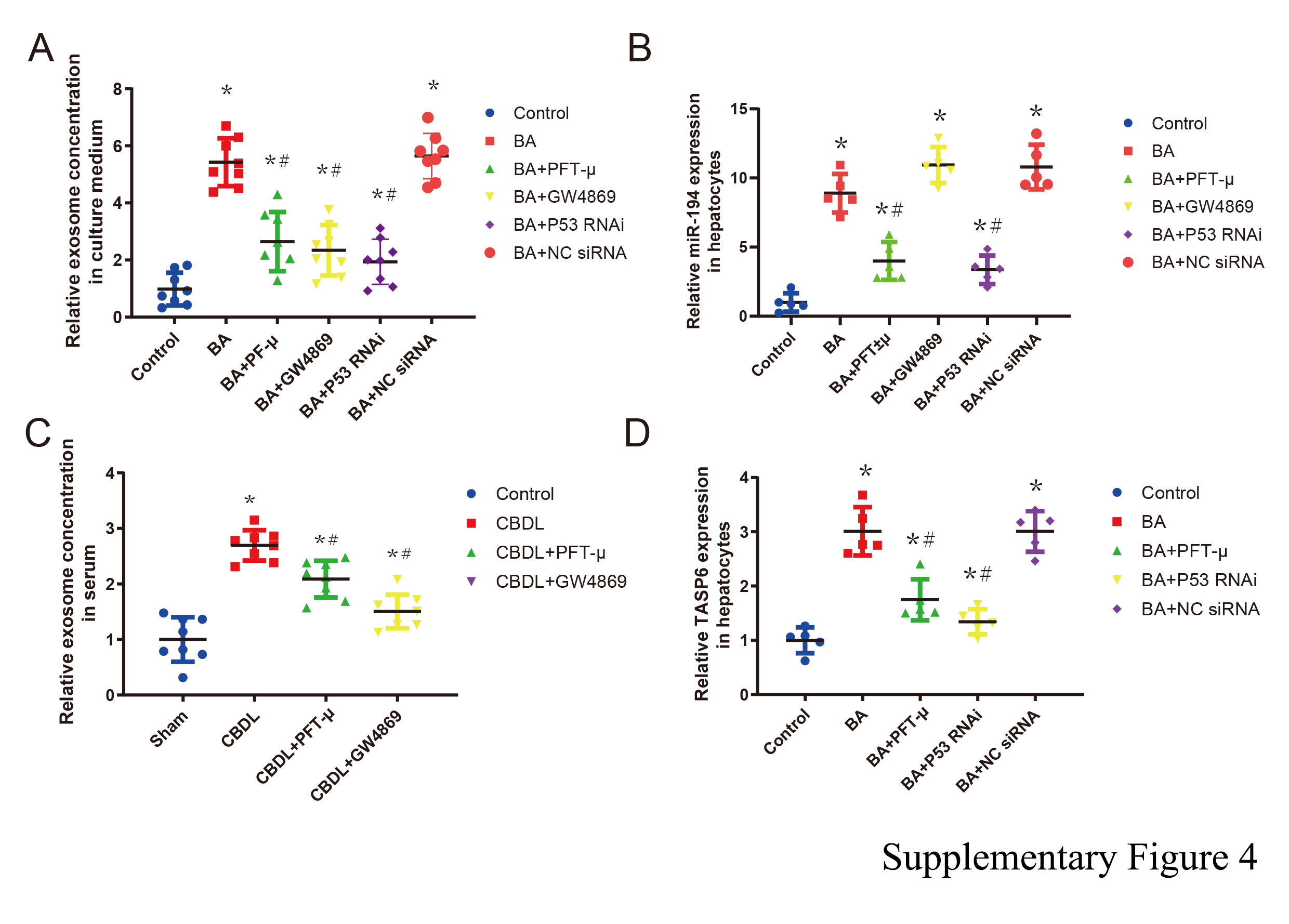

Supplement: Supplementary file 5 — Supplementary Figure 4 [file 41419_2019_2087_MOESM5_ESM.tif]

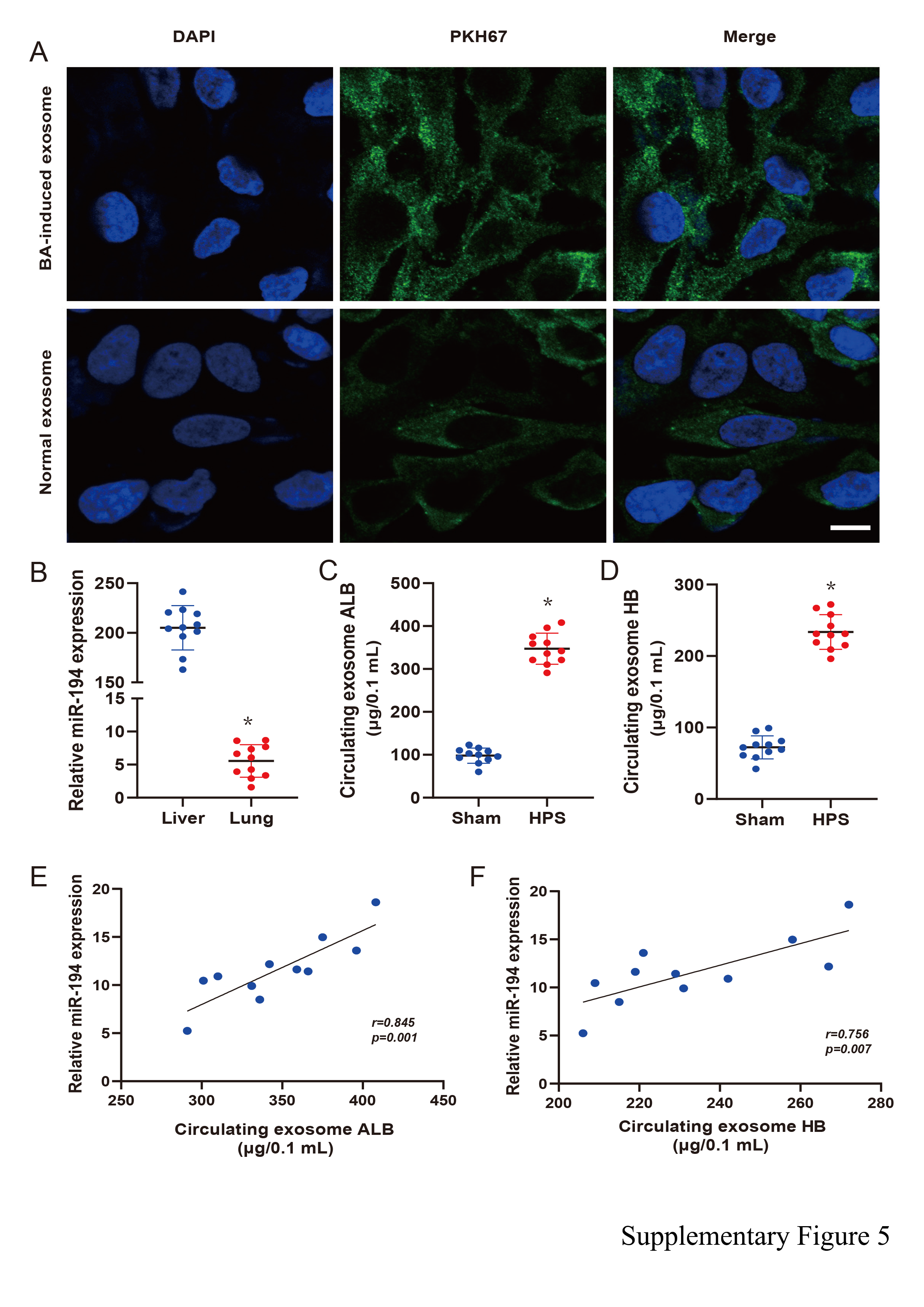

Supplement: Supplementary file 6 — Supplementary Figure 5 [file 41419_2019_2087_MOESM6_ESM.tif]
