# Supplementary material for: Hepatocyte-derived exosomal MiR-194 activates PMVECs and promotes angiogenesis in hepatopulmonary syndrome
Source: Cell Death Dis. 2019 Nov 7;10(11):853. doi: 10.1038/s41419-019-2087-y (PMC6838168; doi:10.1038/s41419-019-2087-y)
Supplement: Supplementary file 10 — Contribution form [file 41419_2019_2087_MOESM10_ESM.pdf]

DECLARATION OF CONTRIBUTIONS TO ARTICLE

ADMC

Manuscript Number: CDDIS-19-2393R

Journal Name: *Cell Death & Differentiation* (the 'Journal')

Proposed Title of the Contribution:

Hepatocyte-derived Exosomal MiR-194 Activates PMVECs and Promotes Angiogenesis in Hepatopulmonary Syndrome (the 'Contribution')

Author(s):

Lin Chen, Yi Han, Yujie Li, Bing Chen, Xuehong Bai, Karine Belguise, Xiaobo Wang, Yang Chen*, Bin Yi*, Kaizhi Lu* (the 'Authors')

For all *CDD* articles, each person named as an author in the published version must be able to show he or she has contributed substantially to the article.

Authorship credit should be based on 1) substantial contributions to conception and design, acquisition of data, or analysis and interpretation of data; 2) drafting the article or revising it critically for important intellectual content; and 3) final approval of the version to be published. Authors should meet conditions 1, 2 and 3.

Any person who cannot be shown to have made a substantial contribution to the article cannot be listed as an author in the final version. The name of any person who is deemed to have made a minor contribution can, however, appear in the Acknowledgments section of the article.

Please complete the table below to indicate the contributions of all named authors to the manuscript.

Author Full Name:	Specification of Contribution to the Manuscript:
Lin Chen	analyzed data;performed the experiments;contributed to writing and prepared figures.
Yi Han	analyzed data;performed the experiments.
Yujie Li	performed the experiments;prepared figures.
Bing Chen	analyzed data;performed the experiments.
Xuehong Bai	analyzed data.
Karine Belguise	analyzed data;performed the experiments.
Xiaobo Wang	analyzed data;performed the experiments.
Yang Chen	designed experiments and helped write the manuscript;contributed to writing and prepared figures;contributed reagents, materials and analysis tools.
Bin Yi	designed experiments and helped write the manuscript;contributed reagents, materials and analysis tools.
Kaizhi Lu	designed experiments and helped write the manuscript.

Please complete the table below to indicate the contributions of all named authors to the figures.

Figure 1:

L.C, W.X. and Y.H. generated the data and prepared image A, D, E, F; L.C and B.C. generated the immune-histochemistry data, the Western blot data and labelled the image B, C, D; Y.L. generated the immunofluorescence data and prepared image G. L.C assembled the figure.

Figure 2:

L.C., X.B. and Y.H. generated the flow cytometric data and prepared image A; X.B. and Y.H. generated the transwell migration assay data and prepared image B; X.B. and Y.H. generated the tube formation assay data and prepared image C; X.B. and Y.H. generated the wound healing assay data and prepared image D, E, F. L.C assembled the figure.

Figure 3:

L.C. and Y.H. generated the microarray data and prepared image A; X.B. and Y.H. generated the qRT-PCR data and prepared B. L.C assembled the figure.

Figure 4:

L.C., X.B. and Y.H. generated the flow cytometric data and prepared image A; X.B. and Y.H. generated the transwell migration assay data and prepared image B; X.B. and Y.H. generated the tube formation assay data and prepared image C; X.B. and Y.H. generated the wound healing assay data and prepared image D, E, G; L.C and B.C. generated the western blot data and prepared image F. L.C assembled the figure.

Figure 5:

K.L. and B.Y. generated the intersection of the TargetScan and AmiGO results data and prepared A; L.C and B.C. generated the western blot data and prepared B; K.B. and X.W. generated the luciferase reporter assay data and prepared image D, E, F; Y.L. generated the qRT-PCR data and prepared image G, H, I. L.C assembled the figure.

Figure 6:

L.C., X.B. and Y.H. generated the qRT-PCR data and prepared image A, B, C, E, F; L.C and B.C. generated the western blot data and prepared D; X.B. and L.C assembled the figure.

In Figure 7, L.C., X.B. and Y.H. generated the immune-histochemistry data and labelled the image A, B, C, D; X.B. and Y.H. collecteded the qRT-PCR data. L.C., X.B. and Y.H. analysised these data from HPS patients and rats. These data were analysised and prepared D, E, F. X.B. and Y.H. prepared image D, E, F. L.C assembled the figure.

Signed for and on behalf of the Author(s):	Print Name:	Date:
kaizhi Lu	Kaizhi Lu	2019.10.16
